# An Epididymis-Specific Secretory Protein HongrES1 Critically Regulates Sperm Capacitation and Male Fertility

**DOI:** 10.1371/journal.pone.0004106

**Published:** 2008-12-31

**Authors:** Yuchuan Zhou, Min Zheng, Qixian Shi, Li Zhang, Wei Zhen, Wenying Chen, Yonglian Zhang

**Affiliations:** 1 Shanghai Key Laboratory for Molecular Andrology, State Key Laboratory of Molecular Biology, Institute of Biochemistry and Cell Biology, Shanghai Institutes for Biological Sciences, Chinese Academy of Sciences, Shanghai, China; 2 Unit of Reproductive Physiology, Zhejiang Academy of Medical Sciences, Hangzhou, Zhejiang, China; 3 Shanghai Institute of Planned Parenthood Research, Shanghai, China; Yale University School of Medicine, United States of America

## Abstract

Mammalian sperm capacitation is an essential prerequisite to fertilizion. Although progress had been made in understanding the physiology and biochemistry of capacitation, little is known about the potential roles of epididymal proteins during this process. Here we report that HongrES1, a new member of the SERPIN (serine proteinase inhibitor) family exclusively expressed in the rat cauda epididymis and up-regulated by androgen, is secreted into the lumen and covers the sperm head. Co-culture of caudal sperms with HongrES1 antibody in vitro resulted in a significant increase in the percentage of capacitated spermatozoa. Furthermore, the percentage of capacitated spermatozoa clearly increased in rats when HongrES1 was down-regulated by RNAi in vivo. Remarkably, knockdown of HongrES1 in vivo led to reduced fertility accompanied with deformed appearance of fetuses and pups. These results identify HongrES1 as a novel and critical molecule in the regulation of sperm capacitation and male fertility.

## Introduction

Sperm maturation or fertilizing capacity are not intrinsic to sperm themselves but they are acquired during their transit through the epididymis after the testis [1]. The epididymis is composed of a long convoluted tube, three main regions are recognized for it, namely caput, corpus and cauda, and the epithelial cells of these specialized epididymal regions create a unique luminal fluid environment by secreting proteins and various fluid components [2]. In this unique and continuously-changing milieu, spermatozoa gradually acquire their forward motility and capacity for fertilization, and then they are stored in a quiescent state in the caudal epididymis. These fluids are particularly well suited to protect spermatozoa from different types of physiological and biochemical aggressions [3]. Although the role of the epididymis in sperm maturation has been well established four decades ago [1], only a few specific molecule of epididymal origin had been demonstrated to be linked to this maturation process [4].

In addition to the maturation in the epididymis, spermatozoa must subsequently undergo numerous membrane modifications before they interact correctly with the oocyte, all of these processes are collectively referred to as capacitation and normally take place within the female reproductive tract [5], [6]. Capacitated spermatozoa subsequently undergo the acrosome reaction (AR) which results in the release and activation of acrosomal enzymes. The sperm cell is then able to bind and penetrate the zona pellucida, and to fuses with the oocyte plasma membrane [7]–[9]. Capacitation comprises a series of processes, such as modifications in sperm surface protein distribution, alterations in plasma membrane characteristics, changes in enzymatic activities and modulation of intracellular constituents [10], [11]. Despite the fact that the phenomenon of capacitation has been discovered over half century ago [5], [12]–[14] and that much progress had been made in identifying sperm events involved in capacitation, few specific molecules of epididymal origin have been identified to be directly involved in this process in vivo. Previously, we reported a newly discovered rat epididymis caudal region specific gene named HongrES1 [15]. The current study reports that, with the aid of sensitive and specific polyclonal antisera against the mature HongrES1 protein, the role of this protein in sperm capacitation had been characterized. Furthermore, by using RNAi in vivo, gene expression knock down of HongrES1 in the rat provides solid evidence that HongrES1 is a novel and critical molecule in regulation of sperm capacitation and male fertility.

## Results

### Native status and localization of the HongrES1 protein in the rat epididymis

**Figure 1:**
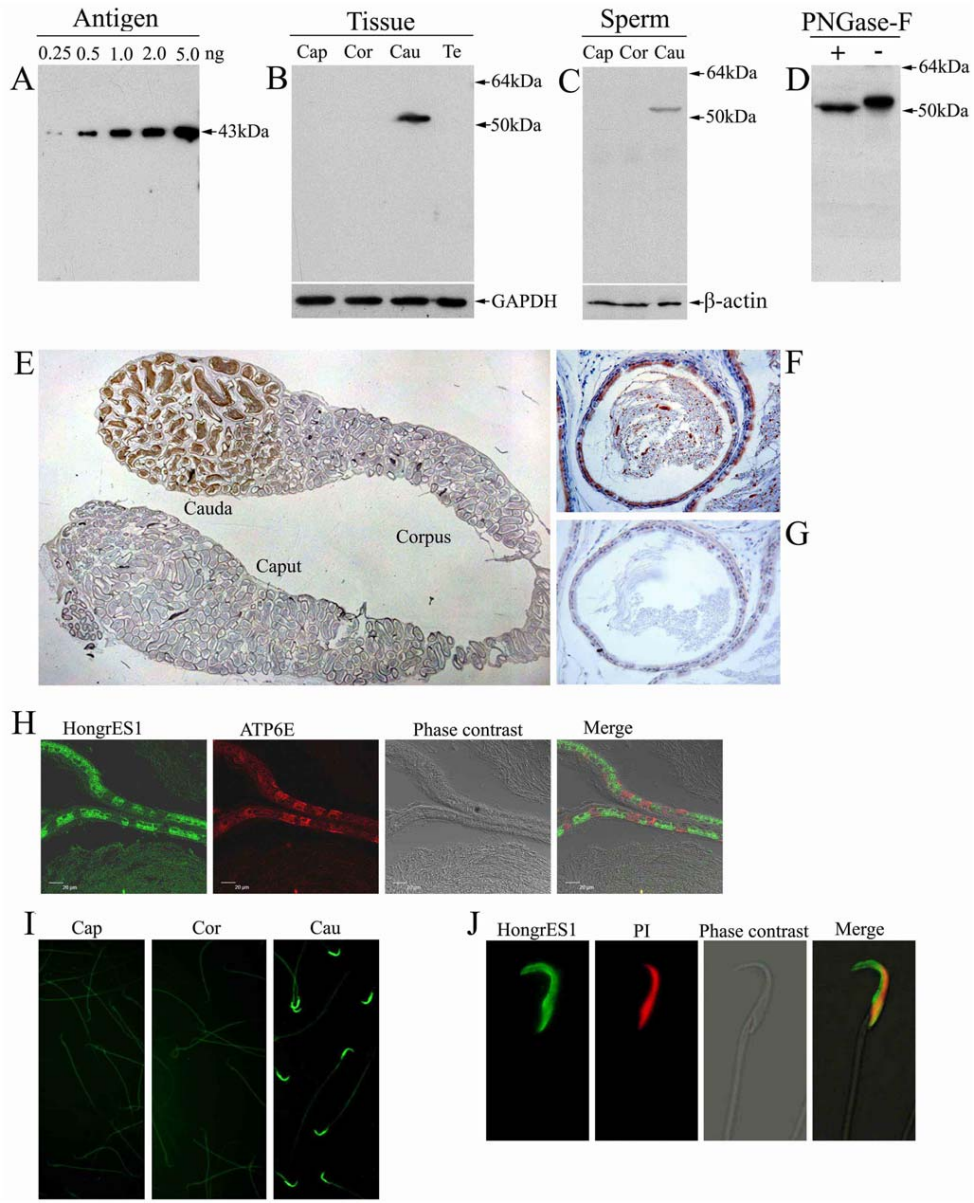
The localization of HongrES1 protein in the rat epididymis. (A) Rabbit polyclonal antisera were raised against HongrES1 recombinant protein (antigen) and the specificity of the antibody towards HongrES1 was verified by Western blotting. (B) Western analysis of HongrES1 protein in total tissue protein extracts from caput (Cap), corpus (Cor) and cauda (Cau) of the epididymis and testis (Te). Blot was probed with monoclonal antibody against GAPDH to assess protein loading. (C) Western analysis of HongrES1 protein in total protein extracts of sperm from caput (Cap), corpus (Cor) and cauda (Cau) regions of the epididymis. β–actin was used for loading control. (D) The change of molecular masses of HongrES1 protein in total tissue protein of cauda epididymis before (−) and after (+) deglycosylation by Peptide N-Glycosidase F (PNGase-F). (E–G) The immunohistochemical staining showed the expression pattern of HongrES1 protein in the whole epididymis (E) and the epithelial cells of the cauda epididymis (F), meanwhile, the immunoreactivity are also detected in the lumen (E and F). Preimmune serum at the same condition showed background level of immunoreactivity (G). (H) The subcellular localization of HongrES1 protein is determined by using cell-specific antibodies. The immunofluorenscence of HongrES1 (FITC-labeled, green) and ATP6E (Rhodamine labeled for clear cells, red) are shown by confocal microscopy. (I) The localization of HongrES1 protein on the spermatozoa by indirect influorescence assays. The positive HongrES1 immunoreactivity is localized the head of sperm from cauda (Cau) region, but not from caput (Cap) and corpus (Cor) regions of epididymis. (J) The HongrES1 binding pattern on the whole head of the cauda sperm. Sperm DNA was stained with propidium iodide (PI) in red color.

We previously showed that HongrES1 cDNA contained 1590 bp nucleotides, with an open reading frame of 1245 bp nucleotides, encoding a 415 amino acid protein with a serine protease inhibitor conserved domain. Northern blot and in situ hybridization assays had indicated that HongrES1 mRNA was specifically expressed in the rat cauda epididymis and restricted to the epithelial cells [15]. To understand the native status and localization of this protein in the epididymis, polyclonal antisera against the recombinant HongrES1 mature peptide was raised successfully in rabbits. The sensitivity and specificity of the antisera were verified by western blot analysis. Figure 1A reveals that even 0.25 ng antigen can be detected by using this antiserum with a dilution of 1∶10,000. Figure 1B shows a distinct single band in the protein extract from the cauda epididymis region but not in those from the caput and corpus, or from the testis. The same band was also found in protein extracts from sperm collected in the cauda epididymis, suggesting that this protein binds spermatozoa (Figure 1C). It was noticed that the size of the band (>50 kDa) was larger than that of the deduced one (45.1 kDa). To account for this difference, the HongrES1 sequence was analyzed using the Prosite software (http://expasy.org/prosite). It was found that there are three N-glycosylation sites (N32, N152 and N380) in the amino acid sequence. After de-glycosylation of the tissue protein extracts by N-glycosidase digestion, a decrease in molecular mass of the HongrES1 band was detected by western analysis (Figure 1D). This result suggests that native HongrES1 protein was glycosylated. Immunohistochemistry assay confirmed that the positive signal was localized to the cauda epididymis and was present also in lumen (Figure 1E, 1F and 1G) and that it was confined to the epithelial cells (green in Figure 1H) but not the clear cells (red in Figure 1H). Indirect immunofluorescence assay further validated that the HongrES1 protein is secreted into the lumen of the cauda epididymis and is bound to sperm (Figure 1I). Figure 1J shows that the protein (green) covered the entire sperm head.

### HongrES1 expression is androgen-dependent and developmentally regulated

**Figure 2:**
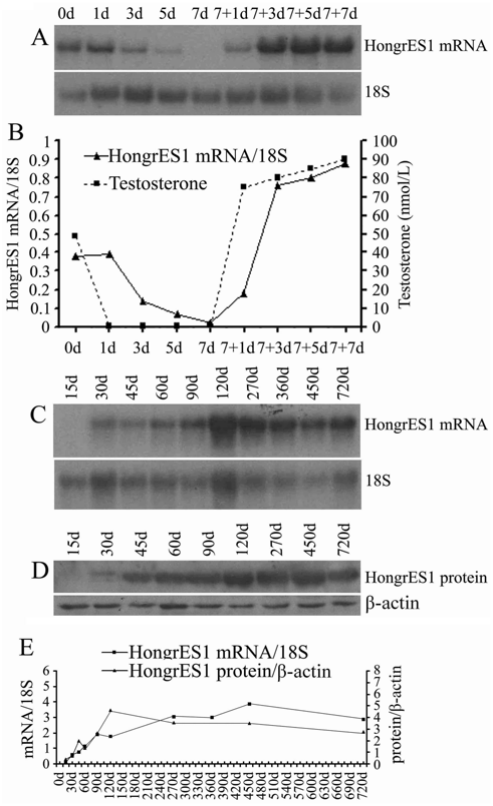
Androgen regulation and developmental change of the HongrES1 expression. (A) Northern blot analysis of adult rat epididymal RNAs from precastration (0 d) and bilateral castration for 1, 3, 5, and 7 days (1 d, 3 d, 5 d, and 7 d) as well as for 1, 3, 5, and 7 days after the initial injection of testosterone propionate applied to the 7 d-castrated rats (7+1 d, 7+3 d, 7+5 d, and 7+7 d). The total RNAs were pooled from four to seven animals at each time-point. (B) The relative expression levels of HongrES1 mRNA (hybridization density of HongrES1 mRNA/18S ribosomal RNA) in the rat cauda epididymis and the serum testosterone level (expressed in nanomoles per liter) during androgen manipulation (n = 4–7). (C) Northern analysis of HongrES1 mRNA and 18S rRNA during development. The samples were collected at 15 d, 30 d, 45 d, 60 d, 90 d, 120 d, 270 d, 360 d, 450 d and 720 d respectively. RNAs were pooled from three animals per group. (D) Western blot analysis of HongrES1 and β–actin proteins during development. The samples were collected at 15 d, 30 d, 45 d, 60 d, 90 d, 120 d, 270 d, 450 d and 720 d respectively. The total proteins were pooled from three animals each group. (E) The relative amounts of HongrES1 mRNA (hybridization density of HongrES1 mRNA/18S ribosomal RNA) and protein (immunoreactivity density of HongrES1 protein/β–actin protein) in the rat epididymis at different developmental stages (n = 3).

Since the epididymis is an androgen-responsive organ [16], the effects of androgen manipulation on HongrES1 mRNA expression were investigated in the castrated rat by northern blot analysis (Figure 2A). In castrated animals, serum testosterone declined rapidly and was almost undetectable from the first day. A clear decrease was found in the HongrES1 mRNA level on the third day post-castration and it was nearly undetectable by day 7 after surgery. Testosterone replacement for the animals 7 days after castration resulted in a rapid increase in the serum testosterone concentration and the HongrES1 mRNA level in the epididymis (Figure 2B). This result indicates that HongrES1 mRNA expression was up-regulated by androgen in vivo. The developmental changes of HongrES1 expression at both mRNA and protein levels throughout the life-span of rats were surveyed by northern blot and western blot analysis (Figure 2C, 2D and 2E). Its expression was detected at day 30 and the high levels were maintained in the aged animals.

### HongrES1 protein is involved in sperm capacitation in vitro

Based on these results, we developed the following hypothesis. As a proteinase inhibitor covering the entire matured sperm head before ejaculation, HongrES1 might protect the sperm head surface proteins from attacked by proteinases in the down-stream tissues or HongrES1 might control the activity of some proteinases on the spermatozoa after ejaculation. An early event confronted by the sperm ejaculated from epididymis is the seminal fluid which contains of various components including a number of proteases [17]. The second event might be undergoing the capacitation and the acrosome reaction in the female tract.

**Figure 3:**
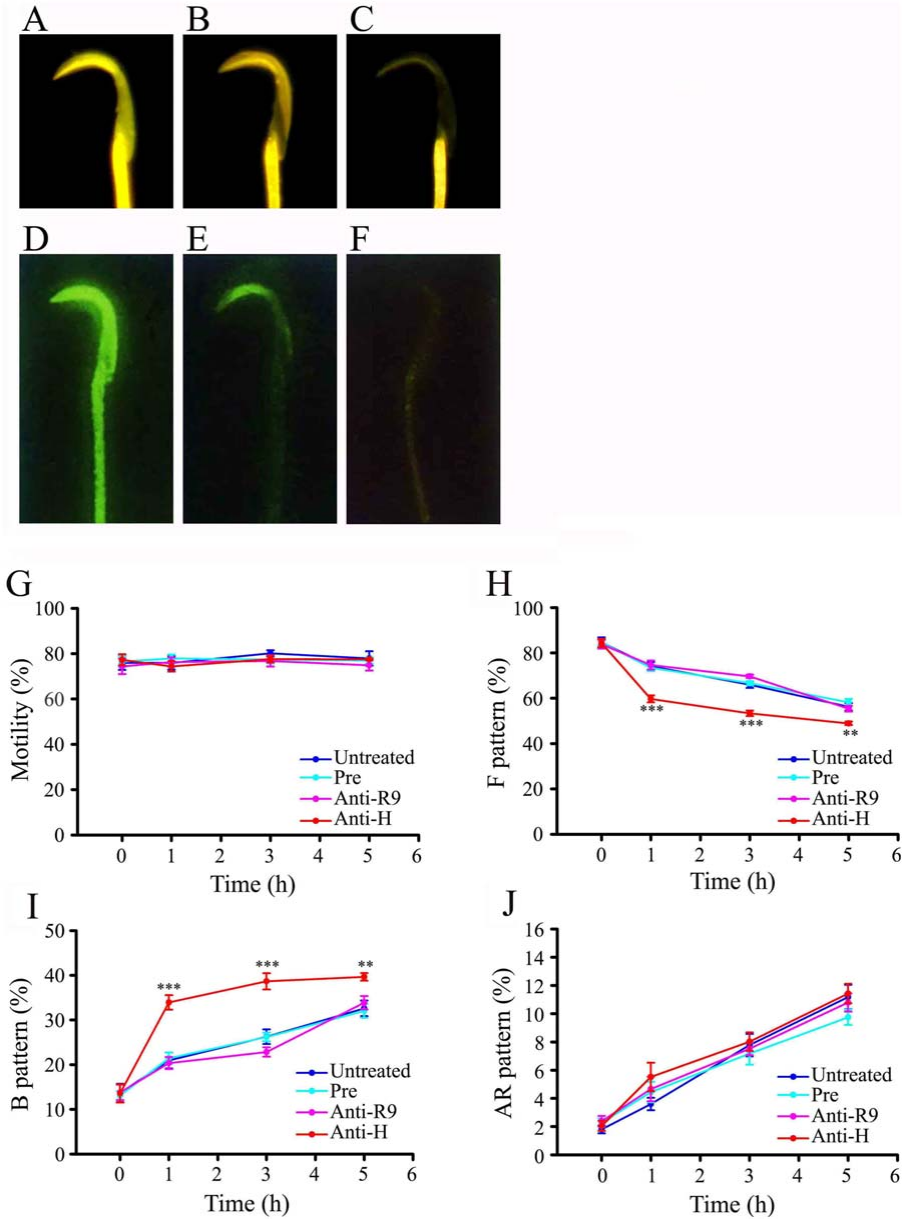
Effect of HongrES1 protein on sperm capacitation and the acrosome reaction in vitro. (A–C) Distinct Chlortetracycline fluorescence staining patterns in uncapacitated sperm with uniform bright fluorescence over the head, F pattern (A), capacitated sperm with a dark band in the postacrosomal region, B pattern (B), and acrosome reacted sperm with dark head except for the tip, which retained some weak fluorescence, AR pattern (C). (D–F) The binding pattern of HongrES1 on the uncapacitated sperm (D), capacitated sperm (E) and acrosome-reacted sperm (F). (G–J) The effect of polyclonal antisera against HongrES1 on sperm state. The changes in the percentage of sperm motility (G), uncapacitated sperm or F pattern (H), capacitated sperm or B pattern (I), and acrosome reacted sperm or AR pattern (J). The treatments with preimmune serum (Pre), HongrES1 antibody (Anti-H), and RNase9 antibody (Anti-R9) were conducted at the time-point of dilution (0 h), and then the aliquots of spermatozoa were taken for recording of motility and CTC assay at 0 h, 1 h, 3 h, and 5 h. In each experiment, at least 300 cells was assessed in duplicate from each sample, the experiment was independently replicated four times, Value are means±SEM (n = 4), **p<0.01, ***p<0.001, as compared with the untreated control.

Since rat semen is not easily collected, we concentrated on the possible role on sperm capacitation and the acrosome reaction by examining changes in vitro. Capacitation can be readily mimicked in vitro by incubation in a chemically defined medium [18]–[21]. This process results in a loss or migration of some proteins on the sperm surface [22]–[24]. In vitro sperm capacitation and the acrosome reaction can be assessed by chlortetracycline (CTC) staining. CTC is a fluorescent antibiotic whose distribution on the sperm changes during the transition from the non-capacitated to the capacitated state and then to the acrosome-reacted state [25], [26], as shown in Figures 3A, 3B and 3C. We therefore followed changes in the sperm binding patterns of HongrES1 protein during capacitation and the acrosome reaction. When cauda epididymal spermatozoa that are bound by HongrES1 were incubated in capacitation medium, the binding immunofluorescence signal attenuated gradually and disappeared (Figure 3D, 3E and 3F). This strongly suggests that changes in HongrES1 protein binding are involved in sperm capacitation and/or the acrosome reaction in vitro. To examine the possible role of HongrES1 during capacitation and the acrosome reaction, the polyclonal antisera were used in this in vitro system. To rule out non-specific interferences, preimmune serum and rabbit polyclonal antisera against RNase9 protein specifically expressed in the caput and corpus of rat epididymis and having no binding activity in the cauda epididymis sperm were used as controls [27]. Figure 3G indicates that none of the treatments (i.e. additions of preimmune serum or specific antisera) affected sperm motility. None of the control sera had any effect on spermatozoa; only spermatozoa treated with HongrES1 antisera revealed significantly changes with the percentage of uncapacitated spermatozoa (F form) significantly decreasing at 1 h, 3 h and 5 h (Figure 3H), and that of the capacitated patterns (B pattern) markedly increasing at the same time points (Figure 3I). The percentage of acrosome reacted spermatozoa (AR pattern) showed no changes (Figure 3J). These results indicate that inhibition of HongrES1 action lead spermatozoa to become more responsive in vitro capacitation and the acrosome reaction system suggesting a possible role for this protein. Since the in vitro system may not entirely reflect changes in vivo we sought to confirm the role of HongrES1 protein in rat sperm capacitation in vivo.

### HongrES1 inhibits sperm capacitation in vivo

**Figure 4:**
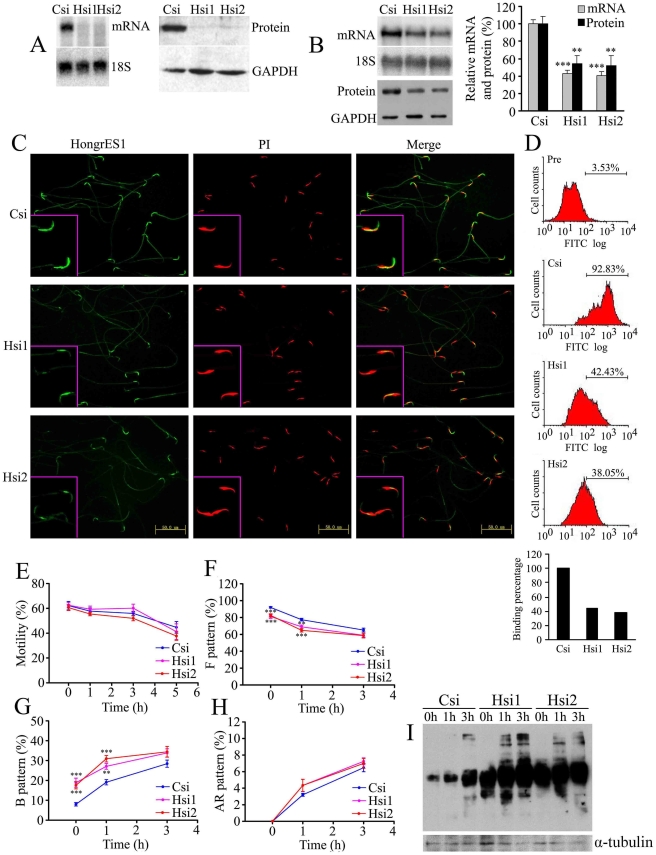
Knockdown of HongrES1 expression by RNAi and its effect of sperm capacitation. (A) Northern blot and western blot analysis showed reduced expression of HongrES1 mRNA and protein in vitro 48 h after siRNAs treatment. Csi, scrambled siRNA control; Hsi1 and Hsi2, two siRNAs specifically targeting the different sites of HongrES1 sequence. 18S and GAPDH were measured as the internal control for RNA and protein loading. (B) The expression of HongrES1 was significantly suppressed by Hsi1 and Hsi2 in rat caudal tissues of epididymis on mRNA and protein levels. Data are expressed as the means±SEM (n = 7–9), **p<0.01, ***p<0.001, as compared with the scrambled siRNA control. Csi, scrambled siRNA control; Hsi1 and Hsi2, two siRNAs specifically targeting the different sites of HongrES1 sequence. 18S and GAPDH acted as a loading control respectively. (C, D) The immunofluorescence staining of sperm from cauda epididymis treated with scrambled siRNA and two specific siRNAs (C), and corresponding binding percentages obtained by flow cytometry (D). The scale bars represent 50 µm in panels. Pre, the negative control of preimmune serum. Csi, scrambled siRNA control; Hsi1 and Hsi2, two siRNAs specifically targeting the different sites of HongrES1 sequence. (E–H) The percentage of sperm motility (E), unpacitated sperm or F pattern (F), capacitated sperm or B pattern (G), and acrosome reacted sperm or AR pattern (H) after expression of HongrES1 was down-regulated. The spermatozoa of caudal epididymis from rats treated by siRNAs for 48 h were collected and incubated, CTC assay was conducted at 0 h, 1 h, 3 h of incubation in the capacitation medium. Data are expressed as the means±SEM (n = 8), **p<0.01, ***p<0.001, as compared with the scrambled siRNA control. (I) The change of protein tyrosine phosphorylation on cauda sperm proteins after HongrES1 expression was inhibited. The spermatozoa of caudal epididymis from rats treated by siRNAs for 48 h were collected and incubated. The spermatozoa total proteins were collected at 0 h, 1 h and 3 h of incubation; α-tubulin was used as loading control. Csi, scrambled siRNA control; Hsi1 and Hsi2, two siRNAs specifically targeting the different sites of HongrES1 sequence.

Gene targeting is a convenient strategy for functional studies, however, gene knock out is not yet practical in rats, so RNAi would be a useful choice. The use of RNAi to knock down gene expression transiently in cell lines has revealed considerable success. Nevertheless, there are still several problems that need to be resolved for gene knockdown in vivo, such as delivery system, how to reach the target cell, or an increase the transferring efficiency [28]. Therefore, only a few successful experiments have been reported but there are few studies for gene function in tissues with blood barriers such as testis or brain [29], [30]. Although the epididymis is a typical blood barrier tissue, the fact that this organ resides outside of the body makes it convenient for local injection procedures. Thus, we decided to use RNAi approach to knock down HongrES1 gene expression in rat epididymis. According to the RNAi mechanism-based rules [31], [32], two siRNAs (Hsi1 and Hsi2) targeting the different sites of HongrES1 gene and a non-silencing siRNA (Csi) as scrambled control were designed and synthesized. Each siRNA with HongrES1 complementary DNA was co-transfected into the mouse epididymal epithelial cell line (PC1), which had no endogenous HongrES1 expression. Figure 4A shows that both siRNAs could inhibit HongrES1 expression almost completely at the mRNA and protein levels at 48 h after transfection. Thus, each effective siRNA was locally injected into the rat cauda epididymis and using in vivo electroporation to improve its transferring efficiency. As shown in Figure 4B, HongES1 gene expression at mRNA and protein levels in the rat epididymis was suppressed by about 50% after 48 h by siRNAs injection as compared with the scrambled siRNA control. Although the injected synthetic siRNAs will be metabolized gradually in the body, this inhibition could still be maintained until 72 h after treatment (data not shown). As expected, the number of spermatozoa bound with HongrES1 protein in the RNAi-treated rats decreased down to about 60% of the scrambled control as shown in Figures 4C and 4D. The reduction in sperm binding led to the significant decrease of uncapacitated patterns and increase of capacitated patterns (Figure 4F and 4G). There were no statistically significant differences in sperm motility and the acrosome reaction observed between treated and control groups (Figure 4E and 4H). The results were identical to those observed in experiments where spermatozoa were treated with HongrES1 antisera. It has been shown that capacitation is characterized by a spontaneous, time-dependent increase of tyrosine and serine/threonine phosphorylation of different proteins [33]–[35], so tyrosine phosphorylation of sperm proteins could be an indicator of whether the capacitation signaling cascade has been activated [36]. Western blot analysis shows that tyrosine phosphorylation of sperm proteins was accelerated when HongrES1 expression was down-regulated by RNAi (Figure 4I). This is consistent with the observations with CTC staining. These in vivo results further directly verified that HongrES1 is a novel molecule of the epididymis origin for the inhibition of sperm capacitation.

### The phenotype of HongrES1 gene knock-down rats

**Figure 5:**
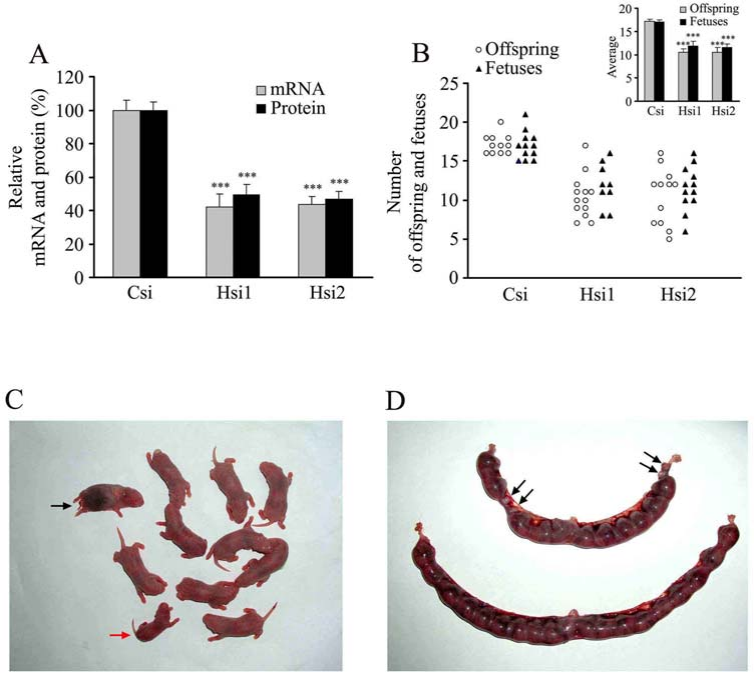
Fertility of HongrES1 knockdown male rat. (A) The relative expression levels of HongrES1 including mRNA and protein in caudal epididymis from all successfully mated rats after RNAi. Data are expressed as the means±SEM. (n = 10–14), ***P<0.001, as compared with the corresponding control. Csi, scrambled siRNA control; Hsi1 and Hsi2, two siRNAs specifically targeting the different sites of HongrES1 sequence. (B) The numbers of live offspring (open cycles) and fetuses (filled triangles) from normal female rats mated with knockdown male rats. Inserts show the average of the results. Data are expressed as the means±SEM. (n = 9–13), ***P<0.001, as compared with the corresponding control. Csi, scrambled siRNA control; Hsi1 and Hsi2, two siRNAs specifically targeting the different sites of HongrES1 sequence. (C) Dead (black arrow head) and small size (red arrow head) individual appeared in the litters after the HongrES1 expression of mated male rats was down-regulated by RNAi. (D) The abnormal embryos (black arrow head) appeared in the uterus after the HongrES1 expression of mated male rats was down-regulated by RNAi.

It is widely accepted that sperm capacitation is a prerequisite for the acrosome reaction and fertilization. We were therefore keen to examine the fertilizing capacity of spermatozoa from rats in which HongrES1 gene expression was knocked down and evaluate the effect of accelerated capacitation that takes place when the apparent inhibitory action of this protein was blocked. After mating, the number of live offspring had a considerable reduction in the litters from receptive female rats that mated with specific siRNA-treated male rats, compared to scrambled-treated control (Figure 5A and Figure 5B open circles). There were some dead (Figure 5C black arrow head) and small-sized pups (Figure 5C red arrow head) in these litters. Table 1 gives the frequency of abnormalities in the litters. To explore embryo development further, implantation of blastocysts in the uterus of pregnant rats was examined. Similar results were found (Figure 5B; filled triangles); i.e., the number of implantated embryos decreased in the group with siRNAs treatment. Noticeably, the decrease was less than that noted when offspring were analyzed. Figure 5D shows an example of implantation in the two groups. There were 19 embryos implanted in the female uterus mated with scrambled siRNA-treated male rat. However, only 15 embryos implanted in the uterus of a female mated with a HongES1 gene knocked down rat sperm. This may be due to abnormalities in sperm-egg interaction or implantation. Moreover, 4 of 15 fetuses appeared to be regressing indicating fetus development failure (Figure 5D black arrow head). Future studies on fetus development should address causes for abnormal development (i.e., reduction in size) and death of offspring. Our in vivo studies further confirm that HongrES1 seem to protect sperm against becoming capacitated before fertilization. An alteration in the timing of capacitation appears to be the cause for fertility reduction and the occurrence of small-sized and dead offspring. This suggests that the usual markers for capacitation, such as CTC staining and protein tyrosine phosphorylation, are insufficient to evaluate the acquisition of a correct capacitation state.

**Table 1:** The frequency of abnormal fertility after HongrES1 knockdown by RNAi in vivo.

	Frequency of dead offspring	Frequency of small size offspring	Frequency of abnormal embryos
Csi	0/11	0/11	0/12
Hsi1	5/13 (1∼2)	1/13 (1)	4/11 (1∼2)
Hsi2	3/12 (1∼3)	1/12 (1)	3/12 (1∼4)

Denominator: the total number of female rats producing offspring or fetuses, numerator: the number of female rats producing abnormal offspring or fetuses, parentheses: the number of abnormal offspring or fetuses in each litter or each uterus corresponding to numerator.

## Discussion

Capacitation represents the completion of sperm maturation that confers on mammalian sperm the acquisition of fertilization-competence either in vivo or in vitro. Capacitation is associated with changes in sperm intracellular ion concentrations, plasma membrane fluidity, changes in metabolism, tyrosine phosphorylation of multiple proteins, and acquisition of a different motility pattern [11], [37]. Although these changes have been known for many years, the molecular basis underlying these events still remains enigmatic. In particular, it is not clear which of the proteins in the epididymis is obligatory for capacitation. The rat epididymis secretary protein, Crisp-1 is the only one reported so far which has a capacitation inhibitory activity in vitro [36]. Here, we provide direct evidence in vivo that HongrES1, a new member of the SERPIN family and specifically expressing in the principal cells of the cauda epididymis, is involved in sperm capacitation. Our results suggest a clear role for HongrES1 protein, spermatozoa would bind HongrES1 on their heads and this protein would act by timing capacitation to take place in the right time and place.

The precisely regulation of capacitation is critical for the normal fertility. Once spermatozoa are capacitated and receive the proper signals or stimuli, they undergo the acrosome reaction rather promptly. On the other hand, the acrosome reacted- spermatozoa survive not long enough and subsequently reduced the ability of fertilization [38]. In vitro capacitation showed that the number of capacitated sperm from subfertile bulls significantly increased compared to fertile bulls at the same timepoint of incubation. These capacitated sperm were able to undergo the acrosome reaction, and subsequent the acrosome reacted-spermatozoa dead doubled [39]. This indicates that prematurely capacitated sperm might result in their premature acrosome reaction and death, whereas, acrosome-reacted cells are no longer able to fertilize, if these phenomena occurs in vivo, it might lead to the decline of fertility. It had been reported that the degree of DNA damage is clearly correlated with the impairment of embryo development and severe DNA damage cause male infertility [40]. Moreover, sperm capacitation could alter the chromatin stability, enhance remodeling asynchrony and result in delayed pronuclei formation and DNA synthesis [41], [42]. Our in vivo results showed that the prematurely capacitated sperm stemmed from knockdown of HongrES1 led to the reduction in fertility and somehow deformed appearance of fetuses and pups. These suggested that HongrES1 play a vital role in elaborate regulation of sperm capacitation.

**Figure 6:**
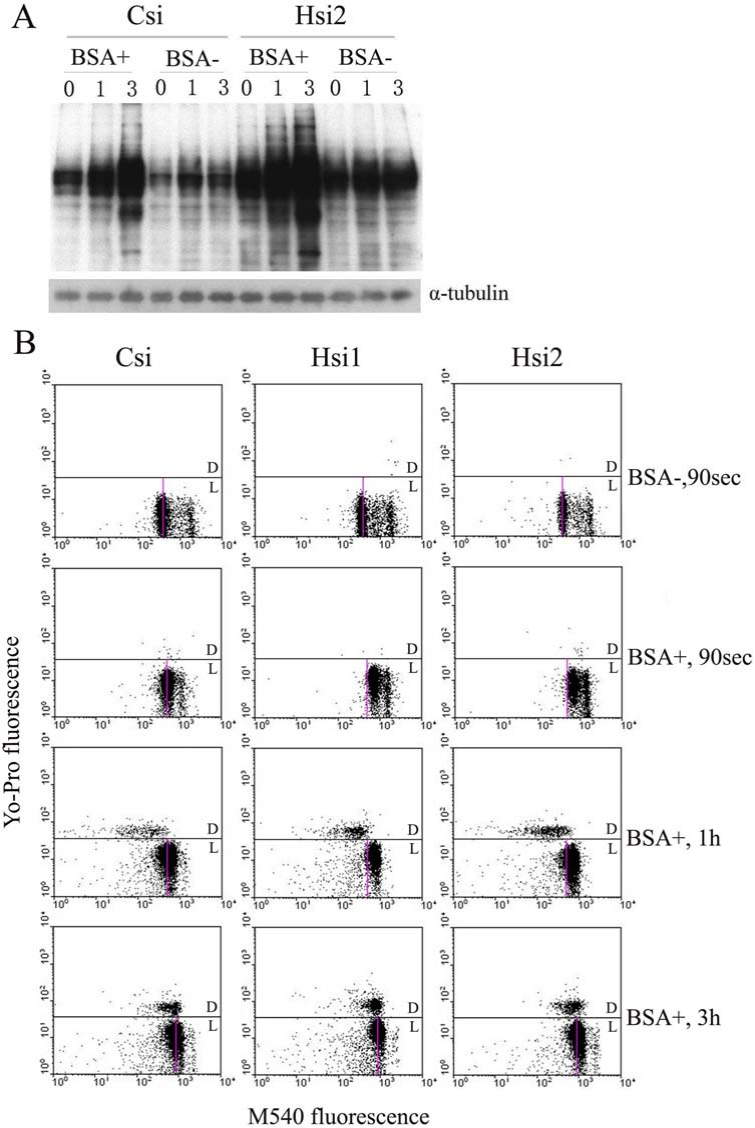
HongrES1 knockdown enlarged the action of BSA on sperm in vitro. (A) The change of protein tyrosine phosphorylation on siRNA-treated sperm after incubation in the presence or absence of BSA. The spermatozoa of caudal epididymis from rats treated by siRNAs for 48 h were collected and incubated. The spermatozoa total proteins were collected at 0 h, 1 h and 3 h of incubation; α-tubulin was used as loading control. Csi, scrambled siRNA control; Hsi2, the siRNA specifically targeting the site of HongrES1 sequence. (B) Flow cytometric dot-plots of merocyanine and Yo-Pro-1 stained sperm population. The spermatozoa of caudal epididymis from rats treated by siRNAs for 48 h were collected and incubated. The spermatozoa were stained and subjected to flow cytometry as described in Material and Methods. D, dead cells (stained with Yo-Pro-1); L, live cells showing merocyanine staining. Csi, scrambled siRNA control; Hsi1 and Hsi2, two siRNAs specifically targeting the different sites of HongrES1 sequence.

Under normal conditions, sperm capacitation take place in the female genital tract, however, the process can be mimicked *in vitro* by incubating epididymal or ejaculated sperm in a defined medium [18]–[21], and a protein source that usually is bovine serum albumin (BSA) which approximates that of oviduct fluid is essential for sperm capacitation [43]. The primary action of BSA is to serve as a sink for the removal of cholestero from sperm plasma membrane [44]. Cholesterol efflux led to changes in the fluidity of the plasma membrane and a subsequent increase in the permeability of the sperm to Ca^2+^ and HCO_3_^−^, the resultant increases in the levels of intracellular Ca^2+^ and HCO_3_^−^ would ultimately stimulate the activity of the sperm adenylyl cyclase, leading to an increase in cAMP and an increase in protein tyrosine phosphorylation. Increase in protein tyrosine phosphorylation is considered to be an indicator that spermatozoa are undergoing capacitation [36], [45]. In our experiment, BSA in the incubation medium is required for normal sperm capacitation and the accelerated sperm capacitation (Figure 6A). Figure 6B showed that knockdown of HongrES1 accelerate the membrane fluidity of sperm treated with BSA and enlarge the action of BSA, this might be the reason why protein tyrosine phosphorylation was accelerated in sperm of HongrES1 knockdown. Sperm capacitation in the female reproductive tract is likely modulated by environmental cues in the luminal fluid, as well as by interactions with oviductal epithelium or other female tissues [46]. Sperm membrane cholesterol efflux might be a result from this interaction, although the identity of such acceptors remains to be clarified. Thus, the membrane fluidity of HongrES1 knockdown sperm may be enlarged and lead to imprecise sperm capacitation in the female reproductive tract, this might result in reduced fertility and deformed appearance of fetuses and pups. At present, it is unknown whether other gene(s) is involved in the process, we need to be excluded.

In HongrES1 gene knocked down male rats, there were mainly three types of spermatozoa, as shown in Figure 4C, i.e. sperm head fully, partially and not totally covered by HongrES1 protein. These differences may be associated to multiple phenotypes such as the decrease in offspring and blastcysts implantation number, the appearance of dead or small-sized offspring and the regressing implanted embryo. It is difficult to isolate spermatozoa either partially- or incomplete-bound HongrES1 to investigate their individual roles in fertilization. Therefore, our future work will concentrate on the generation of HongrES1 conditional knock out mice to reveal the phenotype of pure no HongrES1-binding spermatozoa. In any case, successful knock-down of about 50% of gene expression in the blood-epididymis barrier tissue in vivo, as revealed in this study, will not only lead to studies addressing functions of other genes in tissues with blood barriers such as the epididymis, testis, and brain, but it will also allow for the generation of HongrES1 partially- and incomplete-bound sperm models for a better understanding of events related to capacitation and its timing.

In terms of translational studies, in addition to the evaluation of capacitation, these results are important for the diagnosis of male infertility. Any small molecule to be found repressing expression or inhibiting its binding with spermatozoa could also be developed as a new male contraceptive. Furthermore, if the HongrES1 antibody can be assembled as a capsule to be placed in the vagina before sexual intercourse to mask the HongrES1 sperm binding activity, it might be serve as an emergency contraceptive.

Other questions arise relate to whether HongrES1, like other member of the SERPIN family, have serine proteinase inhibitor activity and why does the epididymis need such a specific proteinase inhibitor. Are there some proteinases specifically expressed in the reproductive tract that serve as its substrates? Moreover, what kind of small molecules can depress its expression or inhibit its binding with sperm? To address these questions, active HongrES1 recombinant protein expression will be a key step in further studies.

## Methods

### Preparation of antibody and immunoblot of HongrES1

An 1161-base-pair cDNA fragment of HongrES1 corresponding the mature HongrES1 peptide of 387 (from 29 aa to the end) amino acids was inserted into a modified PET-28(a) vector (Invitrogen). The recombinant protein was expressed and purified by using a Novagen pET Expression Systems kit (Invitrogen). The polyclonal antisera against HongrES1 were obtained according to our modified immunization methods [47]. Western analysis, immunohistochemical and immunofluorescence staining of tissues, and indirect immunofluorescence detection of protein associated with spermatozoa were conducted following a previously described protocol [27]. Briefly, protein extracts of the epididymal caput, corpus and cauda and spermatozoa from each section were resolved over 12% SDS-polyacrylamide gels, transferred into nitrocellulose membranes, probed with rabbit polyclonal antisera against HongrES1 (1∶10000), the bound IgG was detected with goat anti-rabbit-HRP (Calbiochem, Germany) (1∶10000) and developed using ECL+Plus (Amersham). Protein loading was assayed by probing blots with monoclonal antibody against GAPDH (KangChen Biotech, China) and β-actin (Sigma). Peptide N-Glycosidase F (PNGase-F) (BioLabs, New England) (500 units/µl) was added to the protein extract of the cauda epididymis and the digestion reaction was allowed to proceed at 37°C overnight. N-glycosylation modification of rat HongrES1 was further examined by western blotting. For tissue immunohistochemistry, ABC kit (Sino-American Biotechnology Company) was used for the detection of staining signals according to the manufacturer's instruction. For immunofluorescence, the 1∶800 diluted anti-antisera against HongrES1 and chicken anti-ATP6E IgY antibody (Genway Biotech Inc, San Diego, CA) (dilution 1∶400) were applied, the second antibodies were respectively fluorescein isothiocyanate (FITC)-labeled anti-rabbit IgG (Sigma, USA) (dilution 1∶500) and Rhodamine-conjugated bovine anti-chicken IgY (Santa Cruz) (dilution 1∶200). Sperm were washed out from epididymal caput, corpus and cauda, fixed in 4% paraformaldehyde for 30 min, and immunostained with the polyclonal antisera against HongrES1 (1∶700) and FITC-labeled anti-rabbit IgG (dilution 1∶500). All pictures were taken using a BX51 fluorescence microscope (Olympus) or a confocal microscope (Leica TCS SP2 AOBS).

### Assay for regulation and developmental change of HongrES1

Castration and androgen replacement were conducted following a previously described protocol [27]. Namely, one hundred and twenty day-old normal male Sprague-Dawley rats were castrated bilaterally under sodium pentobarbital anesthesia. Animals were sacrificed on days 0, 1, 3, 5 and 7 after castration as well as 1, 3, 5 and 7 days after the initial testosterone propionate injection. The epididymides were excised and used for RNA extraction. Androgen supplementation began on the seventh day after castration, and rats were injected with testosterone propionate (3 mg/kg body weight) every 2 days. Pooled serum samples for each group were sent to the Shanghai Zhong-Shan Hospital for the measurement of testosterone concentration by RIA. The expression profile of HongrES1 in the rat epididymis during the whole life-span was monitored as previously described procedure [27], [48], the epdidymides of normal male Sprague-Dawley rats were sampled at the age of 15, 30, 45, 60, 90, 120, 270, 360, 450 and 720 days. The tissues from the different developmental stages were measured by Northern blotting and Western blotting.

### Cell culture and siRNAs transfection

Two siRNAs (Hsi1 and Hsi2) targeting HongrES1 sequence and non-silencing siRNA (Csi) for scrambled control were synthesized by Shanghai GeneChem Inc (Shanghai, China). The sense strands of siRNAs are: Hsi1 (sequence 1), beginning at nt308, 5′-CAGGAGCCUUGGAUACUAAdTdT-3′ (sense), Hsi2 (sequence 2), beginning at nt356, 5′-GUAACCUGCUACAUACUAAdTdT-3′ (sense), Csi, 5′-UUCUCCGAACGUGUCACGUdTdT-3′ (sense). PC1 cell lines from proximal caput of mouse epididymis (kindly provided by Dr. Orgebin-Crist) were cultured in a plastic culture plate in the supplemented IMDM containing 10% FCS at 33°C as described elsewhere[49]. The recombinant vector expressing HongrES1 was constructed by cloning HongrES1 open reading fragment into pcDNA_3.1_ vector, then this expression vector and siRNA (mass ratio = 20∶1) were co-transfected into PC1 cells by Lipofectamine according to the instructions of the manufacturer (Invitrogen, Carlsbad, CA). The knock down efficiency was determined by northern blotting and western blotting of extracts from transfected cultures at 48 h after transfection.

### In vivo siRNA treatment by electroporation

The skin covering the testis and epididymis of a male Sprague-Dawley rat (400∼450 g) was cut open under anesthesia, the cauda epididymis was gently squeezed out and fastened with tweezers, and 40 µl of Hsi1, Hsi2 or Csi in PBS solution (75 µg/ml) were injected into the interstitial space of cauda epididymis. Following the injection, in vivo electroporation was used according to the modification of described elsewhere [50]. Briefly, the cauda was grasped between the plates (10 mm diameter) of a pair of tweezertrodes (BTX, San Diego, CA), and 8×50-msec pulses of 50 V were delivered to the tissue using an Electro Square Porator ECM 830 (BTX). This process was repeated in the reverse direction, and the skin was then sutured. The total RNA and protein of cauda epididymis and spermatozoa were collected for assessment 48 h after injection. The animal experiments in this project were conducted according to a protocol approved by the Institute Animal Care Committee. The protocol conforms to internationally accepted guidelines for the humane care and use of laboratory animals.

### Sperm motility analysis

Suspensions of spermatozoa were loaded into flat 100 µm deep microslides (HTR1099, VitroCom Inc., Mt. Lks. N.J., USA) for CASA analysis using HTM-TOXIVOS sperm motility analyzer (Rat Head Toxicology, version 12.3A, Hamilton-Thorn Research, MA, USA). Instrument settings were: temperature, 37°C; objective, ×4; minimum cell size, two pixels; minimum contrast, 80; minimum static contrast, 25; low VAP cut-off, 20.0; low VSL cut-off, 30.0; threshold straightness, 50%; static head size, 0.72–8.82; static head intensity, 0.14–1.84; magnification, 0.80. Thirty frames were acquired at a frame rate of 60 Hz. During analysis, the playback feature was used to check its accuracy.

### Flow cytometry analysis

The percentage of rat sperm with HongrES1 knockdown was analyzed on a flow cytometer (BD-FACScalibur) as described procedure [4]. Sperm were stained with HongrES1 polyclonal antibody at a final concentration of 1∶300, and the secondary antibody, fluorescein isothiocyanate (FITC)-conjugated goat antibody against rabbit IgG (Zymed Laboratories) used at a dilution of 1∶100. We analyzed at least 10,000 individual sperm per sample for FITC fluorescence emissions.

### Evaluation of sperm capacitation

For the assessment of capacitation, sperm from the cauda epididymis were released into the capacitation medium (94.6 mM NaCl, 25 mM KCl, 1.71 mM CaCl_2_, 1.19 mM MgSO_4_, 1.19 mM KH_2_PO_4_, 25 mM NaHCO_3_, 5.56 mM glucose, 10.76 mM sodium lactate and 0.5 mM sodium pyruvate, 0.002% phenol red, 4 mg/ml bovine serum albumin; 50 µg/ml streptomycin sulphate, 75 µg/ml potassium penicillin, pH 7.4, osmolarity about 310 mOsmol/kg). The time-point of spermatozoa dilution was defined as the beginning of capacitation (0 h). Staining with CTC and assess for sperm capacitation and the acrosome reaction were carried out as described elsewhere [26], [51]. After CTC staining and fixation, samples were examined by using BX51 fluorescence microscopy (Olympus) with a ×10 ocular and ×100 objective (oil). The UV light passed through a band-pass filter of 400–440 nm with a relector of 475 nm. In each sample, at least 300 cells were assessed. The tyrosine phosphorylation proteins from spermatozoa were detected by using western blotting. The sperm pellet was suspended in SDS sample buffer, and total sperm protein was performed on PAGE with 8% Tris-glycine gels for western blotting, α-tubulin was used as internal control.

### Sperm membrane fluidity analysis

Cauda sperm from siRNA-treated rats were prepared for membrane fluidity assay using a slight modification of the previously described protocol [52], sperm were released into the capacitation medium containing 25 nM Yo-Pro-1 without BSA. For analysis in the presence of BSA, the collected sperm were diluted in capacitation medium with 2×BSA (8 mg/ml bovine serum albumin). The merocyanine at a final concentration of 1.35 µM was supplemented to sperm suspension; the subsample was then analyzed directly on the flow cytometer (BD-FACScalibur) about 90 sec after addition of merocyanine. The fluorescence of Yo-Pro-1 was detected by the FL1 detector using a 530/30-nm “band-pass” filter, while the influorescence of merocyanine was detected in the FL2 detector using a 620-nm “long-pass” filter. For individual samples, data were collected from 20,000 events per sample.

### Fertility assay

The male rat at 48 h after treatment with siRNA was mated with normal female rats; they were maintained together overnight, vaginal smears of the females were taken in the following morning and examined microscopy; the mated male rat was sampled for determining the knockdown efficiency. The female rats were successfully mated if spermatozoa were present, and then female rats were housed individually. The pregnant females at 18 days were subjected to hysterectomy to determine the number of embryos or fetuses. After birth, the number, viability and the size of offspring in a litter were observed.
